# Genome engineering for improved recombinant protein expression in *Escherichia coli*

**DOI:** 10.1186/s12934-014-0177-1

**Published:** 2014-12-19

**Authors:** Shubhashree Mahalik, Ashish K Sharma, Krishna J Mukherjee

**Affiliations:** School of Biotechnology, Jawaharlal Nehru University, New Delhi, 110067 India

**Keywords:** Recombinant protein expression, *Escherichia coli*, Metabolic engineering, Genome engineering

## Abstract

A metabolic engineering perspective which views recombinant protein expression as a multistep pathway allows us to move beyond vector design and identify the downstream rate limiting steps in expression. In *E.coli* these are typically at the translational level and the supply of precursors in the form of energy, amino acids and nucleotides. Further recombinant protein production triggers a global cellular stress response which feedback inhibits both growth and product formation. Countering this requires a system level analysis followed by a rational host cell engineering to sustain expression for longer time periods. Another strategy to increase protein yields could be to divert the metabolic flux away from biomass formation and towards recombinant protein production. This would require a growth stoppage mechanism which does not affect the metabolic activity of the cell or the transcriptional or translational efficiencies. Finally cells have to be designed for efficient export to prevent buildup of proteins inside the cytoplasm and also simplify downstream processing. The rational and the high throughput strategies that can be used for the construction of such improved host cell platforms for recombinant protein expression is the focus of this review.

## Introduction

**Figure 1:**
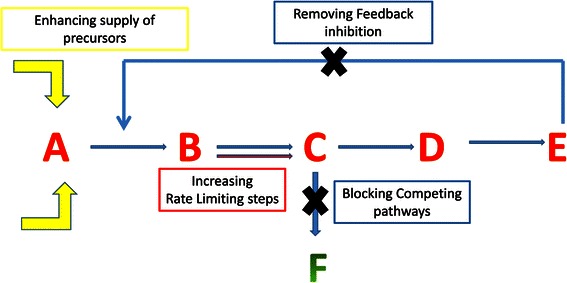
**Metabolic engineering strategies to enhance the flux through a pathway.** The various strategies used to improve the flux from a cellular intermediate to the desired product is shown. This includes enhancing pathways leading to the formation of intermediates (shown in yellow) and pathways which are rate limiting (B to C). Additionally branched chain pathways and feed back controls need to be blocked.

Host cell engineering has emerged as a powerful tool for designing microbial platforms targeted at improved metabolite production. Major successes in this area include improved production of isoprenoids, shikimic acid, isobutanol, amino acids, synthesis of artemesin, lycopene and many such metabolites [1-7]. The basic goal has been to redesign the complete pathway for the biosynthesis of these metabolites by simultaneously engineering multiple steps in the pathway. This has been achieved by a combination of many techniques such as gene knock-ins and knock-outs, promoter engineering, supplementing the expression of critical genes, enzyme engineering and modulation of the regulatory pathways. The commonly used strategies to enhance the metabolite flux through a pathway can be clubbed under the following categories a) Increase the flux through rate limiting steps in the pathway; b) Increase the supply of precursors; c) Block branched chain pathways which lead to by-product formation and d) Remove feedback controls in the pathway (Figure 1).

**Figure 2:**
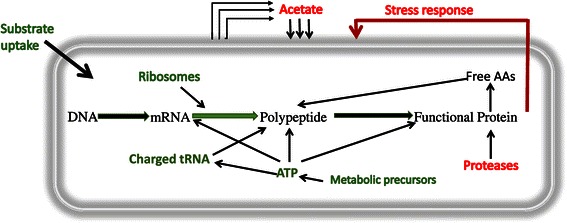
**Simplified schematic of the cellular stress response on various factors affecting recombinant protein synthesis.** The down-regulated pathways are shown in green (substrate uptake, ribosomes, translation rates, tRNA and ATP) and up regulated pathways (Proteases, acetate formation and stress response) are shown in red.

It is possible to extend the same philosophy with minor modifications to help in the design of hosts with improved recombinant protein expression capability. Just like the pathways in metabolite synthesis, recombinant protein expression also involves multiple steps viz. transcription, translation, folding and export. However unlike a typical metabolic pathway these steps are intricately linked to the cellular machinery with multiple host factors determining the flux through each step of the pathway. Hence the cellular physiology and its dynamics have a critical role in determining the overall flux through this pathway. Some important points that can be flagged by this approach are summarized as follows; traditional genetic engineering methods have mostly focused on improving the first step of this pathway i.e. transcription and hence the gains from improved vector design have tended to plateau over time. With strong promoters, the bottleneck in this pathway shifts to the translational step which needs to be up regulated to match the rates of transcription. Otherwise much of the gains of high rates of mRNA synthesis are offset by higher rates of mRNA degradation [8-11]. The supply of precursors which are critical to this pathway are the energy molecules like ATP, amino acids and nucleotides which can become the rate limiting factors in protein biosynthesis. Most importantly recombinant protein expression triggers a cellular stress response which feedback inhibits both growth and product formation, by lowering substrate uptake rates, down-regulating the ribosomal machinery and biosynthesis of ATP (Figure 2). This has a critical impact on the sustainability of the flux through this pathway and typically specific product formation rates decline sharply within a few hours post induction. Since host cell protein synthesis utilizes the same cellular machinery it can be treated as a competing pathway. Thus one way to increase recombinant protein synthesis would be to uncouple growth from product formation, thus allowing the diversion of metabolic fluxes toward product formation. Finally an efficient export mechanism needs to be in place, otherwise there is a theoretical upper limit to which the recombinant protein can accumulate inside the cells. Moreover extracellular expression would significantly simplify downstream processing steps. The challenges associated with designing such host platforms using both rational as well as high throughput strategies is the primary focus of this review.

### Improving transcriptional efficiency

The first step in the pathway for recombinant protein biosynthesis has possibly received the largest attention in terms of improved vector design. A very wide range of vectors are available both for *E.coli* and other microbes with specific features tailored for different applications [12-14]. The rate of mRNA synthesis is determined by both gene copy number and promoter strength, however with strong promoters like the T7 and T5, plasmid copy number has a relatively small role in expression. Rather the use of low copy number, stable vectors allows for lowered levels of ‘leaky’ expression which is important while expressing toxic proteins. Promoter design has thus focused more on titratable and tightly regulated systems rather than strength alone [15-18]. Thus a slow and controlled expression which leads to a properly folded protein can also be an important goal as is obtained with titratable promoters using low inducer concentrations [19,20]. Additionally the use of fusion tags in vector constructs can serve multiple purposes, like efficient purification, improved solubility, increased mRNA stability and more efficient translation [21-27]. To further simplify the process of protein production useful features like auto inducible systems [28-31] and self-cleavable tags have been incorporated in vector design [32-34].

### Removing translational bottlenecks

**Figure 3:**
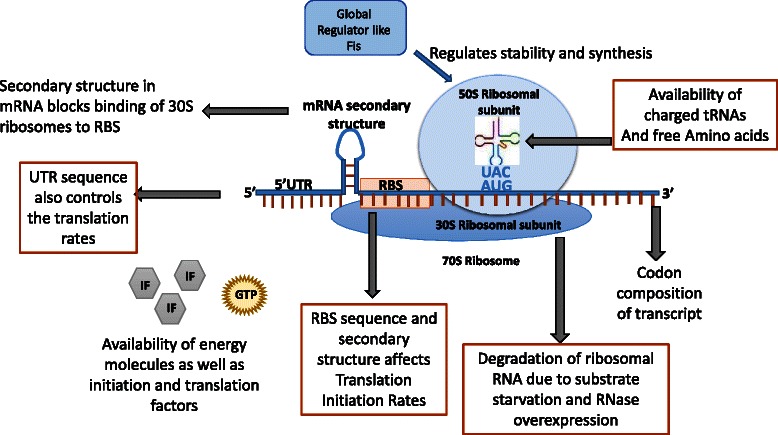
**Cellular factors controlling the rate of translation.** These factors which directly effect translation efficiency are a) mRNA RBS secondary structure affecting the Translation Initiation Rates (TIR) and the UTR sequences, b) Degradation of ribosomes due to substrate non-availability and RNase over expression, c) Availability of charged tRNA which depends on ATP supply and codon bias. Additionally global regulator like FIS also control translation and stability.

Translation has been identified as the rate controlling step in recombinant protein synthesis for most high expression systems (Figure 3). Many factors have a role in controlling translational efficiency including, the first few codons of the mRNA to be translated (translation initiation) and the mRNA secondary structure. The ribosomal binding site (RBS) secondary structure is highly important for efficient initiation of translation. Recombinant protein translation in *E.coli* may be inhibited by presence of secondary structures in the RBS as well as 5’UTR region. Computational tools like ‘ExEnSo’ (Expression Enhancer Software) offer a platform where heterologous gene sequences can be designed on the basis of highest free energy so as to avoid translation inhibition due to mRNA secondary structures. The software also creates a 5’ primer on the basis of the ‘optimized’ sequence which can be used in PCR experiments to amplify the coding sequence of heterologous gene [35]. Similarly a predictive method for designing synthetic ribosome binding sites has been developed which enables a rational control over the protein expression level. This work combines a biophysical model of translation initiation with an optimization algorithm to predict the sequence of a synthetic RBS sequence that provides a target translation initiation rate [36]. Another work involving a random combinatorial DNA sequence library has revealed that not only the SD sequence but the entire UTR sequence, seems to play an important role in the translational process [37] implying that the rate of translation can also be rate-limiting. Translation rate calculators have been designed to estimate protein translation rates based on the sequence of the mRNA and have been shown to give good estimates of the actual level of protein expression [38].

The increase in utilization of the protein synthetic machinery upon induction leads to a degradation of the ribosomal machinery, as a feedback stress response to over expression [39,40] that ultimately leading to a loss in the protein synthesis capacity. This decreased capacity of cells to synthesize proteins, as part of the stringent response, highlights the major challenges regarding the sustainability of recombinant protein production. It has been shown that whereas *E.coli* ribosomes are stable during exponential growth and in the stationary phase, degradation occurs between the transition stages and is independent from the triggering effect of the alarmone ppGpp(p) [41]. Degradation of stable RNA is also associated with conditions of starvation. Thus, depletion of any one of a number of nutrients including phosphate [42], nitrogen [43], carbon [44], or evenMg^+2^ [45] leads to a dramatic loss of RNA. RNase expression is also triggered during the stress response and can contribute to degradation of stable RNA [46].

Rate of translation may also slow down due to non-availability of aminoacylated transfer RNA (tRNA). The availability of charged aminoacylated transfer RNA further depends on codon composition of the transcript. The rationale behind codon usage optimization is to modify the rare codons in the target gene to mirror the codon usage of the host [47,48]. It is also known that the availability of tRNA varies significantly under different growth and stress conditions, which facilitates cellular adaptation to translational dynamics across the genome. Experimental measurements of tRNA concentrations and their charged fractions under stressful conditions have shown that tRNA availability can vary significantly between conditions and over time [49,50]. A computational workflow for estimating codon translation rates based on tRNA availability has been developed. This could be particularly important when considering the over expression of a recombinant protein, where a specific codon composition might lead to the depletion of certain charged tRNA pools [51] or under amino acid limited growth conditions that have been shown to lead to specific charging patterns [52,53]. This deficiency may lead to amino acid mis-incorporation and/or truncation of the polypeptide, thus affecting the heterologous protein expression levels and/or its activity [54]. OPTIMIZER, JCAT, Synthetic Gene Designer, DNAWorks, GeneDesign, Codon optimizer, GeMS are some of the online tools available to optimize codon usage (reviewed in [55]).

Likewise there are models like Ribosomal Flow Model (RFM) which analyses translation process on the basis of its physical and dynamical nature [56]. It considers the effect of codon order on translation rates, the stochastic nature of the translation process and the interactions between ribosomes while predicting the translation elongation step. This approach gives more accurate predictions of translation rates, protein abundance and ribosome densities in comparison to contemporary approaches. Another interesting feature that might be useful for recombinant protein expression and folding is the Translational pause at a rare codon. This provides a time delay to enable independent and sequential folding of the defined portions of the nascent polypeptide emerging from the ribosome [57].

Additionally there are regulatory genes that control the rates of ribosome biosynthesis. CsrA, is a posttranscriptional global regulator that regulates mRNA stability and translation, which in turn is regulated by two sRNAs *csrB* and *csrC* [58-60]. The *E.coli* DNA binding protein Fis is a transcriptional modulator involved in the regulation of many cellular processes, including the activation of rRNA synthesis. High-level expression of *fis* in early, mid, or late log cultures has been shown to result in growth phase and medium-specific variations in cell growth, rRNA synthesis, and ribosome content [61].

### Improving energy availability

The synthesis of recombinant proteins is energy intensive and interferes with the host physiology [62]. The high energy demand during recombinant protein production leads to an enhanced need for ATP generation at the cost of biomass formation [63]. This increases maintenance energy requirements which manifests itself as an increased metabolic burden on the cells [64]. In order to sustain this energy demand, cells take up alternative pathways like substrate level phosphorylation leading to acetate formation by carbon overflow metabolism. As a consequence of reduced biomass formation, excess NADPH might be converted to NADH via the soluble transhydrogenase, filling the electron transport chain for additional ATP generation. This hypothesis is supported by a positive correlation between ATP production and productivity, while an inverse correlation exists between biomass yield and productivity. Although the ATP generation rate increases with increasing demand, the TCA cycle activity remains constant, indicating a limited capacity of the TCA cycle to overcome the postulated metabolic burden [65].

It has been shown that protein synthesis consumes approximately two-thirds of the total energy produced by a rapidly growing *E.coli* cell [66]. Consequently, much effort has been focused on understanding the mechanisms of ATP and GTP usage during protein synthesis. It was thus observed that phosphoenolpyruvate carboxykinase (PCK) when expressed in *E.coli* under glycolytic conditions helped in increasing the intracellular ATP levels, leading to enhanced protein production, of both the model proteins GFP (intracellular) and Alakaline Phosphatase (extracellular) [67]. Polymerization of amino acids as well as aminoacyl-tRNA synthetase requires large portion of ATP to mediate amino acid-charged tRNA synthesis [68]. It is known that the concentration of aminoacylated-tRNA (charged tRNA) molecules is higher in rapidly growing bacteria, and it has been postulated that the availability of the charged tRNA is one of the check points that determines the rate of protein translation [69].

### Cofactor regeneration

Cofactors play an important role in generation of correctly folded, stable and functional recombinant proteins [70]. Any imbalance in cofactor consumption and regeneration can lead to a severe reduction of growth. Since these are the driving forces behind most anabolic pathways as well as oxidative phosphorylation, it is necessary to design strategies to enhance cofactor regeneration. OptSwap is a computational method which predicts strain designs by identifying optimal modifications of the cofactor binding specificities of oxidoreductase and complementary reaction knockouts [71]. Another mathematical framework, cofactor modification analysis (CMA), is a well-established constraints-based flux analysis method for the systematic identification of suitable cofactor specificity engineering (CSE) targets while exploring global metabolic effects [72]. Several genetic strategies employed for cofactor engineering have been reviewed earlier [73-76].

### Facilitating protein folding and export

A major effort in recent years has focused on improved protein folding *in vivo* using chaperone co-expression [40,77-79]. These molecular chaperones essentially belong to Hsp70 chaperone family. Thus DnaK which is an Hsp70 homolog binds to unfolded hydrophobic stretches and helps protein folding while chaperones like GroEL encapsulates the nascent polypeptide and prevents inter molecular interactions [80]. A major issue is matching the availability of chaperones with the rates of production of the nascent polypeptide to prevent misfolding. This is a problem when high level expression systems are used. The only way to circumvent this is to have lower but sustained rates of protein expression leading to a slow buildup of the recombinant protein. Another important aspect is providing an oxidizing environment for correct disulfide bond formation in the cytoplasm or catalyzing bond formation of the oxidized protein in the periplasm [81]. This has been attempted by introducing genes for formation of disulfide bonds. Thus strains capable of producing properly folded proteins, even those with multiple disulphide bonds, are now available [82-87]. An *E.coli* strain has recently been designed for protein transport which oxidizes disulfide bonds in the cytoplasm and then efficiently exports these disulfide containing proteins using a signal peptide. These test proteins include alkaline phosphatase (PhoA), a phytase containing four disulfide bonds (AppA), an anti-interleukin 1bscFv and human growth hormone [88].

**Figure 4:**
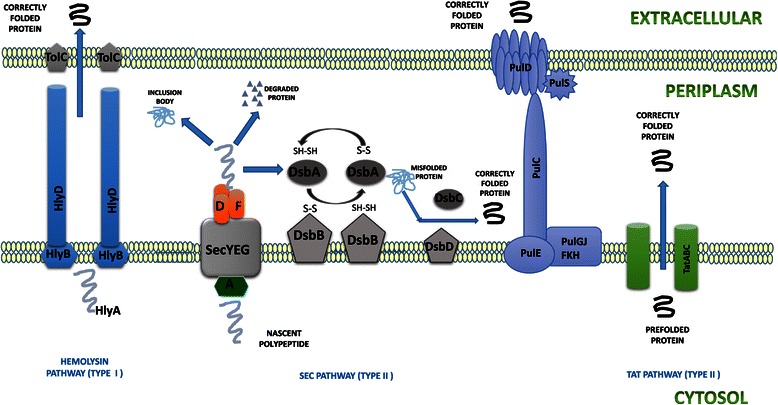
**Various pathways for protein translocation (Type I and II).** The type II Sec-dependent pathway is the most commonly used and gets overloaded leading to accumation of mis folded proteins in the cytoplasm. However the TAT dependent pathway is used for export of folded proteins and thus requires proper folding in the cytoplasm itself.

The more challenging task is protein export not just to the periplasm but to the extracellular medium. This would not only greatly simplify purification but also remove the upper bound on the accumulation of proteins in the culture. There are five pathways for protein secretion in *E.coli* Type I, II, III, IV and V. However, only the first and second secretion pathways are commonly used in recombinant protein secretion. Type I pathway directly targets proteins from cytoplasm to extracellular medium [89,90]. Studies have shown that the Type II Sec dependent pathway gets overloaded leading to an accumulation of unfolded proteins [91-95]. Plasmid based over expression of SecY, SecE and SecG proteins, which are the major interacting partners of SecA, resulted in a strong enhancement of a) translocation ATPase activity, b) preprotein translocation, c) capacity for SecA binding, and d) formation of the membrane-inserted form of SecA [96] (Figure 4). There are reports of a few proteins which get naturally secreted into the medium [97-99]. Others like GFP which do not get secreted through the Sec dependent pathway have been successfully exported via a modified TAT dependent secretion pathway [100]. In another work synthetically designed lipase ABC transporter domains (LARDs) from *P.fluorescens* lipase were attached to GFP and epidermal growth factor (EGF). The fused proteins were successfully secreted with the ABC transporter and showed lipase activity as an intact fused form in the supernatant [101]. These examples highlight some of the important developments in this area which has the potential of making *E.coli* into a truly secretory protein expression system.

### Feedback inhibition of product formation

It is well known that growth rates decline post induction in most cultures. It was earlier postulated that this was due to the ‘metabolic burden’ associated with the diversion of metabolic fluxes towards recombinant protein synthesis [102]. However a careful analysis of experimental data shows that this rate of decline of growth post induction, in the absence of substrate limitation is an intrinsic property of the cell and specific to the protein being expressed [103]. Thus some proteins like γ –interferon [104], α-interferon [105] even when they are expressed at high levels do not adversely affect growth while others like insulin [106], GMCSF [107,108], streptokinase [109], lead to a complete growth stoppage post–induction. Interestingly this retardation is also dependent on whether a protein is expressed as inclusion bodies or as a soluble protein like GMCSF and streptokinase [110-112]. Therefore attempts to explain growth retardation in terms of the amino acid composition of the expressed protein has limited predictive value [51,113,114] though clearly different amino acids impose different metabolic requirements on the cell. Clearly a static “diversion of fluxes” model is inadequate to explain this growth retardation rather a complex cellular dynamics controls both the growth and product formation kinetics.

This phenomenon of growth retardation is better understood by analyzing the cellular stress response to recombinant protein expression, which characteristically depends on the nature of protein, the form of expression (whether soluble or inclusion bodies) and the level of expression (whether from a strong or weak promoter) [115,116]. Moreover environmental factors such as the medium composition (presence or absence of complex nitrogen sources) [19,84,117,118] and the specific growth rate may also effect the nature of this response [119,120]. Studies have shown that this stress response mimics the features of the heat shock response, the oxidative stress response and the stringent response [39,114,121]. There are a set of common genes which get up or down-regulated due to this response which belong to the substrate uptake, amino acid and ribosomal biosynthesis genes and those involved in energy metabolism [39,121]. Others which are specific to the form of expression, like IB expression are *clpBP*, *dnaJK*, *groLS*, *grpE*, *hslRUV*, *htpGX*, *ibpAB*, *lon*, *rlmE*, *rpoD*, *yrfI* [122-124]. It is difficult to model this stress response using systems biology tools like Flux Balance Analysis (FBA) to predict the changes in fluxes of various pathways [125,126]. This is primarily because the commonly used metabolic model of *E.coli* with the largest set of pathways covers only ~30% of genes which are actually present in the organism [127,128]. Most of the differentially expressed genes are not part of this metabolic network and this unavailability prevents their expression mapping and FBA analysis using the model. Thus the stress response is better modeled as the triggering of key regulatory genes which in turn trigger a cascade of other downstream genes [121]. Efforts have been made to develop regulatory models which can analyze the complex interplay of the regulatory and metabolic networks [129-132]. These models could be applied in the analysis of the stress response due to recombinant protein over expression and provide us with leads for designing improved expression platforms. However there are as of now very few published reports on attempting to modulate this stress response by gene knock-ins or knock-outs. One would expect that knock-out of non-essential genes which get up-regulated due to the stress response or conversely supplementing gene expression of the down-regulated genes may help alleviate this stress and have a beneficial effect on recombinant protein expression [133,134].

The use of metabolic engineering strategies to remove the bottlenecks in recombinant protein production identified by analyzing this stress response has helped in improving the supply of precursors like NADPH, modification of global stress regulators and increasing the flux of the down regulated metabolic pathways including that of substrate uptake [135-140]. Thus increasing glycerol uptake by over expressing the *glpK* gene, lead to a 35% higher rhIFN-β expression as compared to control cultures [133]. The issue of acetate formation has been solved by knocking out genes (*ackA*, *pta*, *ppc* and *poxB*) in the acetate biosynthesis pathway leading to improve the recombinant protein production [135,138,140]. Supplementation of down regulated genes either through plasmid based expression or chromosomal integration have been successfully tried, e.g. Expression of the *zwf* gene coding for glucose-6-phopshate dehydrogenase in the Pentose Phosphate pathway helps to provide building blocks like nucleotides and NADPH and thus improves recombinant protein expression [139]. Knock out of ppGpp as well as the deletion of the global regulator *rpoS* (which is triggered by ppGpp) has been shown to enhance the recombinant protein expression [141-144]. The metabolic engineering strategies to improve the *E.coli* phenotype for recombinant protein production has also been reviewed earlier [145].

### Uncoupling growth from product formation

The growth associated nature of recombinant protein production means that high specific growth rates need to be maintained in order to get high specific product formation rates. Since product concentration in a bioreactor is determined both by biomass concentration and specific product yield, we have the twin requirements of growing cells to high cell densities while simultaneously maintaining high specific growth rates. Together these requirements usually lead to oxygen or heat transfer limitations in the bioreactor especially during scale up. Hence the ability to produce product at high rates using slow growing or non-growing cells could greatly simplify the bioprocess strategy for high level product formation. Secondary metabolites are a very good example of how the non-growth associated nature of product formation kinetics allows the easy separation of growth and product formation phases in a bioreactor. In the case of recombinant protein synthesis, we need to ensure that the resting cells are metabolically active in terms of substrate uptake and energy metabolism as well as transcription and translation.

One interesting development in this regard was the Quiescent cell expression system where growth and product formation kinetics were decoupled [146]. Growth stoppage was achieved by over expressing a small RNA ‘Rcd’ which blocks cell division. However recombinant protein expression is unimpaired, and since the translational machinery is not required for biosynthesis, these cells have a significantly higher productivity compared to normally growing cells [147]. Further studies on the mechanism of Rcd showed that it binds to tryptophanase leading to the overproduction of indole [148]. Thus an exogenous supply of indole was also able to block cell division without affecting recombinant protein expression. However indole targets multiple sites in the cell [149-151] and may not be a preferred option for recombinant protein expression. Therefore targets downstream of indole which specifically blocks cell growth without affecting metabolic activity needs to be identified in order to achieve improved quiescence.

### Tools for host cell engineering

This section deals with the vast array of techniques that have now become available, greatly simplifying the task of host modification to obtain the desirable phenotype by rational or high throughput approaches.

#### Single gene modification strategies

While the use of plasmid based methods for supplementing gene expression may be useful in ‘proof of principle’ studies, they have severe limitations. There is an upper limit to the number of target genes that can be supplemented; also the level of supplementation may be far higher than desired, leading to an unnecessary metabolic burden on the cells. Thus chromosomal engineering which leads to the construction of plasmid-less, marker-less strains has the advantage of extending the practical exploitation of the modified hosts in industry [152]. Also promoter engineering allows us to fine tune the expression of genes to desired levels [153,154]. One of the earliest strategies to design Single-gene knockouts was using the λ RED-ET system. Here the gene to be knocked out is replaced with an antibiotic resistance gene, usually kanamycin or chloramphenicol. If required, the selection marker can be removed by expressing the Cre or FLP recombinases that acts on the FRT or loxP site that is present in the flanking region of the selection marker or antibiotic cassette [155-161]. Another commonly used method for Single-gene knockouts is the P1-mediated transduction [162-166]. This method has gained popularity because of the availability of the Keio library of single gene knock-outs of non-essential genes in BW25113 which can be easily transferred into almost any *E.coli* strain [167]. Many researches prefer to use the vector plasmid pKO3 which integrates into the chromosome by homologous recombination creating tandem duplication at the non-permissive temperature. When shifted to the permissive temperature, the presence of the pSC101 replication origin in the vector ensures that it is excised from the chromosome. The presence of the *sacB* gene from *B.subtilis* in the vector allows us to screen for the loss of the vector sequence by growing the cells in the presence of sucrose [168].

The main limitation of these techniques is that they can be applied for single gene modifications and if multiple knock-ins and knock-outs have to be done, then these have to be done sequentially in a time consuming manner.

#### High throughput genome engineering methods

**Figure 5:**
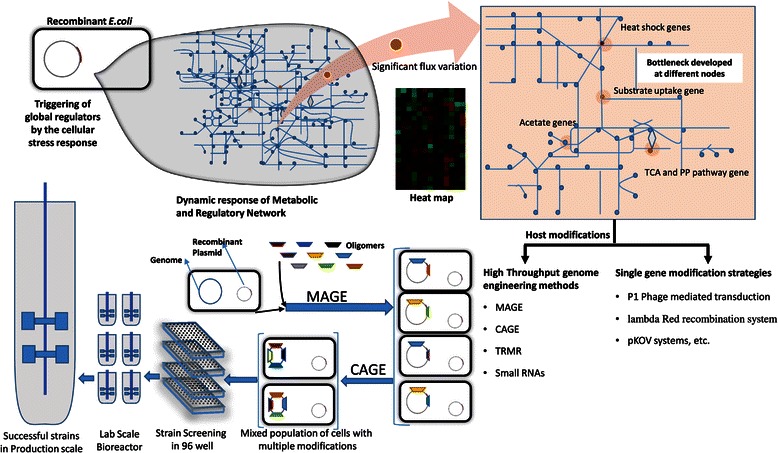
**Overall Strategy for host cell engineering using rational and high throughput methods.** Leads obtained from analysis of cellular physiology and Omics studies can be used to identify system level bottlenecks. These can be addressed by both single gene modifications or high throughput methods to generate improved platforms for expression. These are initially tested in microbioreactor format to select the best clones for further scale-up.

High throughput methods have been developed for genome engineering like Multiplex automated genome engineering (MAGE), Trackable multiplex recombineering (TRMR) and use of small regulatory RNAs [169-172]. These methods can create simultaneous random combinatorial modifications in the *E.coli* genome. Till now these approaches have mainly been used for evolutionary studies and pathway optimizations in *E.coli*. The same strategy can be applied for improving recombinant protein expression. As MAGE, works through oligonucleotide-mediated allelic replacement in an iterative manner it is capable of introducing multiple modifications in different locations of genome. Therefore several oligomers can be designed to perform multiple modifications iteratively, which can help in identifying the combinations which lead to the desired phenotype including that of enhanced protein capability (Figure 5). Recently, a group led by Y.S. Ryu, has modified MAGE so that it is not restricted to EcNR2 strains of *E.coli* [160]. This is important since it is well known that there is a wide variation in the expression levels obtained with different *E.coli* strains [173]. Also strains carrying different modifications and having the desired phenotype can be combined in a step wise fashion using Conjugative assembly genome engineering (CAGE) [174,175]. This approach can be used to look for synergy between various modifications. The major drawback with MAGE is that it also accumulates unwanted off-target mutations [175] and thus a method for genome engineering at multiple locations with greater precision needs to be developed [176]. Another method for rapid modification of many genes in *E.coli* is TRMR. This technique uses a large number of synDNAs with multiple desirable sequence features, to modulate the expression levels of genes. The synDNA contains different RBS which replaces the native RBS and a Molecular barcode is used to track the allele in mixed populations [177]. Another novel approach for high throughput metabolic engineering is the use of a transcriptional vector to express small chromosomal DNA fragments of *E.coli* itself. Since some fragments get inserted in the opposite orientation, they act as an anti-sense RNA and create a library of down-regulated pathways which can be screened for improved recombinant protein expression [178]. This has been extended to the use of synthetic Small regulatory RNAs (sRNAs) which helps in the modulation of gene expression [169]. This method is useful when we need to down regulate gene expression rather than completely knocking-out the gene, making it an indispensable tool for studying the effect of essential genes on cellular phenotype.

#### High throughput screening strategies

The screening of a large number of gene knock-in and knock-outs to select the desirable phenotype of improved expression capability is time consuming. The simplest approach to screen a very large number of clones is to use FACS based screening for cells expressing fluorescence tagged proteins like GFP [179,180]. Thus libraries with engineered genomes can be screened for the highest producers by using appropriate sorting protocols [181,182]. However such modified hosts may not necessarily over express other proteins, given the very specific nature of host-protein interactions. Another strategy would be the selection of quiescent phenotype, in order to uncouple growth and product formation. For this one can screen for a growth stoppage phenotype which typically leads to elongated cell morphologies due to stoppage of cell division [183]. Simultaneously or later these cells can be checked for recombinant protein expression capability after growth arrest. Such techniques can be coupled with automated devices where cultures can grow in 96 well plate formats. Such technologies have proven to work well in clone screening and help in quickly identifying the best performers from a large number of clones e.g. BioLector from m2p Labs [184,185], Bioscreen C from Oy Growth Curves Ab Ltd [186-190], Clone Screener from Biospectra AG and the Ambr reactor from TAPBiosystems. Apart from growth profiling, these systems can also do online monitoring of fluorescence, pH, dissolved oxygen and NADH [185] and are reviewed in [191-195].

## Conclusion

The complex linkages between cellular physiology and the multiple steps in recombinant protein synthesis makes the task of removing bottlenecks in this pathway a difficult exercise. However we now have a wealth of information from transcriptomic, proteomic and metabolomic studies on the cellular factors affecting this pathway as well the changes in flux to this pathway due to the cellular stress response. The data has been useful in rational design of host cells with better expression capabilities. Also the use of high throughput screening methods have allowed us to, reverse engineer these desired phenotypes, adding vastly to the repertoire of beneficial knock-ins and knock-outs. With the development of tools for genome scale engineering to generate multiple knock-ins and knock-outs we can now study the synergistic response of these changes which were earlier limited to one or two modifications. These could lead to major improvements in the design of host platform for high level recombinant protein expression.
